# Organophosphate Poisoning: Demographics, Severity Scores and Outcomes From National Poisoning Control Centre, Karachi

**DOI:** 10.7759/cureus.8371

**Published:** 2020-05-31

**Authors:** Anum Amir, Asif Raza, Talha Qureshi, Ghulam B Mahesar, Salman Jafferi, Farhan Haleem, M Ali Khan

**Affiliations:** 1 Internal Medicine, Civil Hospital, Karachi, PAK; 2 Internal Medicine, Jinnah Postgraduate Medical Centre, Karachi, PAK; 3 Surgery, Jinnah Postgraduate Medical Centre, Karachi, PAK; 4 Gastroenterology, Jinnah Postgraduate Medical Centre, Karachi, PAK

**Keywords:** poisoning, organophosphate, demographics, severity scoring, peradeniya organophosphorus poisoning (pop) scale, outcomes

## Abstract

Introduction

Organophosphate ingestion is the commonest cause of self-harm encountered at poison control centers in Pakistan. It usually affects a young populous. Organophosphates are found in various forms and formulations that are easily accessible to the general public. These compounds are extremely potent poisons causing rapid clinical deterioration with minimal ingestion or exposure.

Signs and symptoms can range from mild or none to severe such as bradycardia, miosis, fasciculations, seizures and altered level of consciousness. Poisoning severity is measured using the Peradeniya Organophosphorus Poisoning (POP) scale. Mortality rates are relatively low for mild to moderate disease. Severe disease as calculated by the POP carries an exceptionally high mortality rate.

The National Poisoning Control Centre (NPCC) at Jinnah Postgraduate Medical Centre, Karachi treats an extraordinary number of poisoning cases on a daily basis. Despite this data pertaining specifically to OP ingestion is nearly absent. There have been no studies analyzing the various aspects of organophosphate poisoning in the last 30 years to the best of our knowledge. Here, we look to rectify this.

Aims

To evaluate the demographics, severity scores and outcomes of organophosphate poisoning cases in the last year from the NPCC, Karachi.

Methods

This was a retrospective study. It was held from 1^st^ January 2019 to 31^st^ December 2019. All data was recorded from patients admitted to the NPCC with a proven diagnosis of organophosphate poisoning.

Results

Three thousand and three hundred patients were inducted into this study. Over 3/4th of the patients were teenagers or aged less than 30 years. Almost all referrals were made from within the city. Overall survival rate at 28 days was 89.45%. Most patients presented with mild to moderate disease as calculated by the POP; severe disease had a mortality rate of nearly 50%.

Conclusion

Organophosphates make up a significant portion of all cases of poisoning treated at the NPCC. The POP is an excellent tool to evaluate disease severity. Overall survival rates are good but mortality rate is high for severe disease even in young patients.

## Introduction

Organophosphate (OP) ingestion is one of the most common emergencies treated at poisoning control centers worldwide. They are irreversible cholinesterase inhibitors that are effective in very low concentrations. Sign and symptoms include but are not limited to increased bronchial secretions, seizures, weakness, neuropathy and miosis [1]. Respiratory failure is the most common cause of death in OP poisoning.

OPs are capable of causing death within minutes of exposure. Mortality depends upon the type of compound ingested, amount ingested, route of ingestion, general health status of the patient and prompt diagnosis. Favorable outcomes are seen with early diagnosis, administration of atropine along with intravenous fluids and proper respiratory support including oxygen therapy [2].

OPs are used as insecticides, drain cleaners, rat killers, pesticides and germicides [3]. All of these products are easily available in the market. They are cheap and do not require back ground checks or any scrutiny for purchase. Even once purchased the said items are not kept in secure locations at home or work places. Easy accessibility makes them the poison of choice. All of this leads to high mortality rates with estimates ranging from 5% to 35% [4].

The National Poisoning Control Centre (NPCC) at Jinnah Postgraduate Medical Centre, Karachi sees an inordinate amount of cases each year. Nearly half of these cases are due to OP poisoning and almost all have suicidal intent or intentional self-harm. Here we analyze the demographics, length of stay, types of OP used and clinical outcomes of OP poisoning in the last year.

## Materials and methods

Study design and location

This was a retrospective study. It was held at the National Poisoning Control Center (NPCC), Medical Unit I, Jinnah Postgraduate Medical Centre, Karachi.

Induction criteria and duration

All patients admitted to the NPCC from 1st of January 2019 to 31st of December 2019 (one year). Patients with valid Medico-legal numbers and proven diagnoses of organophosphate poisoning were inducted into the study. All data were retrieved from the Records section, NPCC, Karachi, Pakistan. Cases analyzed in this study are a part of the justice/legal system. Information such as treatment regimen given, suicidal or homicidal intent and other illnesses could not be accessed for this study and were off limits. Patient confidentiality was made certain. Non-probability and consecutive sampling technique was used in this study.

Primary outcome

Mortality rate at 28 days was the primary outcome; mortality rates with respect to subtype of OP poisoning were also analyzed. Outcomes with respect to severity of OP poisoning were also evaluated.

The Peradeniya Organophosphorus Poisoning (POP) scale

Severity of OP poisoning was calculated using the Peradeniya Organophosphorus Poisoning (POP) scale (Table 1). In the POP scale, six parameters are examined and given a score of 0, 1 or 2 depending upon the findings. Scores are added up and severity ascertained. A score of 0-3 indicates mild poisoning; a score of 4-7 shows moderate poisoning and a score of 8-11 demonstrates severe poisoning.

**Table 1:** The Peradeniya Organophosphorus Poisoning (POP) scale

Parameter	Criteria	Score
Pupil Size	>2 mm	0
	<2 mm	1
	Pinpoint	2
Respiratory Rate	<20/min	0
	>20/min	1
	>60/min	2
Heart Rate	>60/min	0
	41-60/min	1
	<40/min	2
Fasciculation	None	0
	Present, generalized/ continuous	1
	Both generalized and continuous	2
Level of consciousness	Conscious and rationale	0
	Impaired response to verbal command	1
	No response to verbal command	2
Seizures	Absent	0
	Present	1
0-3: mild poisoning, 4-7: moderate poisoning, 8-11: severe poisoning		

Data analysis

Statistical Package for Social Sciences (SPSS) version 21 (IBM Corp., Armonk, NY) was used to analyze the data.

## Results

Three thousand and three hundred patients were admitted to the NPCC during the one-year study period with proven diagnoses of OP poisoning. This constituted approximately one-third of all admissions to the NPCC for the year of 2019.

Demographics

Patients were generally very young or young adults, with a mean age of 24.10 ± 9.44 years. The youngest patient was aged 7 years at the time of admission. There was a slight female preponderance among the patients. Almost all patients were referred from within the city. Demographics are shown in Table 2.

**Table 2:** Demographics

	N = 3300
Gender	
Male	1518 (46.0%)
Female	1782 (54.0%)
Location	
Karachi	3231 (97.90%)
Non-Karachi	69 (2.10%)
Age groups	
≤18 years	1128 (34.18%)
19-29 years	1452 (44.0%)
30-39 years	453 (13.72%)
40-49 years	165 (5.0%)
≥50 years	102 (3.69%)
Overall Age (mean)	24.10 ± 9.44 years

Organophosphate poisoning types

The most common form of organophosphate ingested were the off labels products followed by rat killers. The different types of organophosphate poisoning are summarized in Table 3.

**Table 3:** Organophosphate poisoning types

	N = 3300
Off label products	1239 (37.54%)
Rat killers	960 (29.09%)
Drain cleaners	516 (15.63%)
Insecticides	468 (14.18%)
Phenyl	117 (3.54%)

Primary outcome

There were a total of 198 expiries during the study period. Overall mortality rate was 6.0%. Mean length of stay was 2.17 ± 1.80 days. Primary outcome and length of stay are given in Table 4. Relative mortality of each subtype of organophosphate poisoning is given in Table 5.

**Table 4:** Primary outcome

Outcome at 28 days	N = 3300	Length of stay at hospital (days)
Survived	2952 (89.45%)	2.12 ± 1.65
Expired	198 (6.0%)	4.27 ± 3.39
Left against medical advice	150 (4.54%)	0.78 ± 2.13

**Table 5:** Relative mortality of each type of organophosphate poisoning

	Mortality (%)	Relative mortality within the poisoning type group (%)
Off label products	71 (2.15%)	5.73%
Drain cleaners	50 (1.51%)	9.68%
Rat killer	39 (1.18%)	4.06%
Insecticides	26 (0.78%)	5.55%
Phenyl	12 (0.36%)	10.25%

Disease severity scores

The Peradeniya Organophosphorus Poisoning (POP) scale was used to calculate disease severity. Over two-thirds of the patients had mild disease. The mortality rate was lowest for mild disease and highest for severe disease. Severity scores with their relative mortality rates are summarized in Table 6.

**Table 6:** Disease severity scores with their respective mortality rates

	N = 3300		
Severity*	28-day survival	Expired	LAMA**
Mild disease	2074 (62.84%)	2 (0.06%)	137 (4.15%)
Moderate disease	526 (15.93)	25 (0.75%)	13 (0.39%)
Severe disease	352 (10.66%)	171 (5.18%)	Nil
*Severity of the poisoning was calculated using the Peradeniya Organophosphorus Poisoning (POP) scale (Table 1); ** LAMA = Left against medical advice			

## Discussion

OP poisoning is a disease of the young, affecting both genders equally [5]. Such was the case with our study. The mean age of the patients was 24.10 ± 9.44 years. Almost 80% of the patients were younger than 30 years. The most predominant age was 18 years with 153 cases. In general all ages in the teenage bracket had the highest number of cases. Less than 10% of cases were aged 40 years or above.

This is a complex matter as to why such a young populous opts for such a drastic measure. This choice is influenced by social inequities, peer pressure, pop culture, financial disparity, lack of opportunities, fear of missing out, family problems, educational misadventures or failures and matters of the heart to name a few. It is beyond the scope of this study to integrate and evaluate these aspects. However, such an insight into OP poisoning is much needed in Pakistan.

Almost all referrals were made from within the city. While Karachi is one of the largest cities in the world and houses a diverse group of people, the ambulance network in the city is far spread, inexpensive and very easily accessible. The same can be said about the secondary care centers that usually first receive the patient and give first aid. Both of these factors contributed to the relatively low mortality rates seen in this study.

The type of OP poison ingested greatly influences the outcome. The commonest type of OP ingested were the “Off label products”. This is consistent with previous data [6]. These are readily available, cheap, locally made and are not registered or regulated. Most of these are available in liquid form in small glass bottles. They are used for various purposes and were the go-to poison for most patients due to factors discussed here. The concentration and potency of these compounds are questionable.

Rat killers and insecticides are available in powdered, semi solid and liquid states. Majority of the patients ingested the liquid form. Similarly drain cleaners and phenyl are only available in liquid or semi solid forms. It is the opinion of the authors that irrespective of the OP available to the patients, liquid forms represented an easier ingestible option that was readily available. Liquid forms were also associated with increased mortality (see below).

Survival rate was high in this study. Early referrals from within the city, adequate first aid, reduced potency of the “off the label” products, early administration of atropine and oxygen when indicated all contributed to this. With the exception of the most severe cases, arrival times (since the ingestion of the poison) to the emergency department were short. This greatly contributed to survival rates and reasons for it are discussed later.

Perhaps the most pertinent factor for improved survival was the mild disease status of the patients at presentation as calculated by the POP scale. The POP scale was first introduced in 1993 and analyzed many variables on admission [7]. Two variables, i.e. the need for ventilatory support and administration of atropine in the first 24 hours of admission, were associated with increased mortality rates. Patients with mild disease (by far the largest group) in our study did not require either.

As to why so many patients presented with mild disease status is a matter of opinion. Almost all OP ingestion is suicidal. However, we believe that most of the time this is a cry for help; a desperate measure to seek attention. There are stark differences between disingenuous suicidal attempts and genuine ones which are all too apparent at presentation. Majority of the patients are of the earlier group.

True suicidal intent has certain characteristics. Patients are often found in isolation, late and with severe disease having ingested heavy doses. There is no relay of message of intent at all. Such cases are far few and in between. On the contrary, all of these characteristics are absent with disingenuous attempts. Patients generally declare what they have done or ingested, are easily found, take minimum possible doses, are cooperative during treatment and relay messages of intent to a relative or friend(s).

While overall mortality was low, the in-group relative mortality for two subtypes of OP poisons was approximately 10% i.e. drain cleaners and phenyl. Both of these products are manufactured by multinational brands as such the potency and effectivity of these products is guaranteed for their intended purposes. This very fact makes them lethal poisons.

Furthermore, large quantities of these two poisons are available in the house primarily in liquid form. On average a drain cleaner or phenyl bottle contains 500-1000 ml of fluid. Thus, at the very onset a large amount of poison is available for ingestion. Because of the nature of the products these are usually kept in the bathroom, a private space with freedom to ingest considerable amounts. All of these factors contributed to the relatively high mortality rates seen with drain cleaners and phenyl.

As previously discussed, a huge proportion of the patients presented with mild to moderate disease as assessed by the POP scale. Not only did these patients had very low mortality rates but their median stay at the hospital was one day; a short period by all means. Some of these patients chose to leave against medical advice. Perhaps the lack of symptoms gave them a false sense of security. Nothing can be said with certainty as to what made them make this decision.

One in ten patients had severe disease. Nearly all mortality in this study can be attributed to this group of patients. Patients with severe disease had a staggering mortality rate of 48.57%. Median duration of stay at the hospital was five days in these patients. Symptoms such as bradycardia, dehydration, miosis, altered level of consciousness and respiratory complications require extensive management and need days and at time even weeks to ameliorate.

The POP scale was found to be an excellent tool for gauging response to treatment and overall prognosis of the patients. It is purely a symptomatic scale and forgoes the need to calibrate the dose of the poison, route of ingestion or potency of the poison. All of these factors are important to prognosticate patient outcomes, but it is not always possible to determine these objectively.

The outcomes for patients with respect to mild, moderate and severe disease when calculated by the POP in this study were comparable to previous data [8]. Severe disease had the highest mortality; most cases of severe disease had respiratory complications. The scale is also dynamic. Development of altered level of consciousness or fasciculations or seizures was ominous and portended a guarded prognosis. Many a patient with these signs eventually required mechanical ventilatory support.

Shortcomings

The study did not record or analyze the dose of the ingested poison(s), the reasons for ingestion, comorbids, previous attempts of self-harm, history of substance abuse, use of other therapeutic medication, psychiatric illnesses or history, addictions, criminal history, socioeconomic status, educational history, or employment (or unemployment) records.

Patient follow-up was limited to 28 days only. Lab parameters such as hemoglobin, white blood cells, platelets, hematocrit, mean corpuscular volume, urea, creatinine, liver function tests, international normalized ratio, bleeding times, iron profile and nutritional analysis were not available for the purposes of this study. Radiology was omitted all together.

## Conclusions

Three thousand and three hundred cases of OP poisoning were admitted to the NPCC in the year 2019. It primarily affected young people without gender bias. The POP was a useful tool in prognosticating OP poisoning outcomes and management. Mild to moderate disease had an excellent survival rate at 28 days. Nearly half of the patients with severe disease died within the same period.
